# Associations between religiosity/spirituality with insulin resistance and metabolic syndrome in the Midlife in the United States (MIDUS) study

**DOI:** 10.1371/journal.pone.0319002

**Published:** 2025-02-21

**Authors:** Kevin S. Masters, Caitlyn L. Wilson, Jennifer Morozink Boylan

**Affiliations:** 1 Department of Psychology, University of Colorado Denver, Denver, Colorado, United States of America; 2 Department of Health and Behavioral Sciences, University of Colorado Denver, Denver, Colorado, United States of America; Emory University, School of Public Health, UNITED STATES OF AMERICA

## Abstract

Religiosity and spirituality (R/S) are central aspects to the lives of many people worldwide. Previous research suggests a potentially beneficial relationship between R/S, mostly understood as religious service attendance, and mortality. Though important, this research often fails to account for the complex and multidimensional nature of R/S. Also lacking is an adequate understanding of the physiological mechanisms that may link R/S with mortality and other health outcomes. Insulin resistance and metabolic syndrome, subclinical physiological processes that are influenced by the types of lifestyle factors and psychological factors that R/S addresses, serve as two possible biological mechanisms linking R/S and health outcomes. This study investigated the relations of R/S, defined as service attendance, support from one’s religious community, and composite variables comprised of several diverse R/S indicators, in relation to insulin resistance and metabolic syndrome both cross-sectionally and in longitudinal analyses across 8–10 years in the Midlife in the United States (MIDUS) study. Results, controlling for important covariates (demographic factors, self-rated health, chronic conditions, depressive symptoms for all analyses; diabetes status and body mass index for insulin resistance analyses; antihyperlipidemic medications for metabolic syndrome), demonstrated nonsignificant relationships for all measures of R/S and both insulin resistance and metabolic syndrome in both cross-sectional and longitudinal analyses. Integrating these findings into the limited research on physiological mechanisms in the R/S and health relationship suggests that the area lacks consistent findings. Additional studies that use heterogenous, representative samples and further refine the operationalization of R/S are indicated.

## Introduction

Religiosity and spirituality (R/S) are central aspects of the lives of many people worldwide influencing how they behave, what they believe to be true about the world, with whom and how they socialize, and how they experience emotion [1]. Recent research confirms that, in the United States and worldwide, religious belief is common and more than 70% of populations in countries in Africa, the Middle East, South Asia, and Latin America indicate that religion is “very important” in their lives [2]. Further, several decades of scientific research demonstrate that some aspects of R/S, often measured as religious service attendance, are associated with lower all-cause mortality risk across a wide range of populations including samples from Asia, Europe, South America, and North America [3–20]. The mortality risk reduction associated with service attendance ranges from 10 to 40% across studies with an overall average point estimate of about 25% [14]. Given the considerable size of the relationship with mortality combined with the prevalence of exposure, many aspects of R/S may be powerful social determinants of health [19].

Research on religious service attendance and mortality provides a foundation upon which to study R/S and health but is insufficient. First, R/S is inherently a complex, multidimensional phenomenon that is not adequately captured by only considering attendance at religious services. Second, the underlying processes that could account for relationships between more frequent service attendance and lower mortality risks have not been thoroughly examined. Third, the existing literature on R/S and health and mortality is mixed, with several studies failing to support the hypothesis that R/S is uniformly beneficial for health [21–24]. Finally, less research has considered whether, and to what extent, dimensions of R/S affect physiological dysregulation, quality of life, or morbidity outcomes in addition to mortality risks in the general population. Though some investigations have demonstrated that additional measures of R/S beyond service attendance predict a variety of health outcomes [9,10,19,21], more research is needed. The Midlife in the United States [25] study is important in this regard as it provides repeated measures of religious identification, spirituality, R/S coping, private religious practices, daily spiritual experiences, R/S based mindfulness, religious support, and frequency of religious service attendance. It also contains measures that allow for the calculation of potential physiological mechanisms including insulin resistance and metabolic syndrome (MetS), the foci of this investigation. Thus, this significant longitudinal dataset provides a relatively unique opportunity to assess several aspects of R/S and explore possible R/S and health relations in the general population in a more nuanced way.

Propositions regarding how R/S might influence health outcomes focus on behavioral, social, psychological, and physiological mechanisms. Specifically, self-control, emotional self-regulation (e.g., mood control, refocusing awareness) and behavioral self-regulation (e.g., ability to limit unhealthy behavior or engage in healthy behavior) are likely pathways whereby R/S may affect health and mortality risk [26,27]. For instance, some major R/S traditions provide faith-based guidance on health behaviors (e.g., moderate or no alcohol consumption, no use of tobacco, regular social support, regular physical activity, healthy or vegetarian diet) that align with current lifestyle recommendations for maintaining or improving health (e.g., the American Heart Association’s Life’s Essential 8 [28]). Further, for some individuals, R/S beliefs and practices offer benevolent ways of understanding the events of the world and individuals’ lives. Practices such as prayer, meditation, and receiving comfort from ritual, may provide coping with psychological and emotional stress, which may likewise promote healthier physiological functioning and lower morbidity risks. Illustrating the role of physiological dysregulation in R/S and mortality relations, Bruce and colleagues (4) demonstrated that allostatic load (i.e., an index of dysregulation across multiple physiological systems) partially mediated the relationship between service attendance and mortality over 14 years in the NHANES III (1988-1994) cohort. The authors suggest that these effects may be due to lifestyle and stress reduction properties associated with some components of R/S, though the properties themselves were not studied.

Cardiometabolic disorders like insulin resistance and MetS are likely subclinical pathways, or mechanisms, that may connect some aspects of R/S to morbidity and mortality. Insulin resistance is a physiological condition whereby the body’s cells become less responsive to the effects of insulin, a hormone that is essential for the regulation of blood sugar levels and facilitation of the uptake of glucose into cells, primarily muscle and fat. MetS is an increasingly common and complex medical condition characterized by the presence of at least three of five cardiometabolic risk factors including abdominal obesity, hypertension, elevated triglycerides, low high-density lipoprotein (HDL) cholesterol, and elevated fasting glucose. The prevalence of MetS has grown, in eight years, from about a quarter of the US population to now being present in approximately one-third of US adults [29]. The condition is more common in older adults with over 50% of adults over age 60 meeting criteria [30]. The relationship between MetS and insulin resistance is significant, complex, and bidirectional with factors related to the onset and increase in severity of both conditions potentially exacerbating each other. Insulin resistance, however, is often viewed as a central component of MetS, linking the various criteria for the syndrome in a positive feedback loop or “vicious cycle”. Both conditions are exacerbated by lifestyle factors (e.g., physical inactivity, poor dietary quality, psychological stress) and both predict the onset of type 2 diabetes mellitus (T2DM) and cardiovascular disease [31–33]. Given that both outcomes are influenced by the types of lifestyle factors that some R/S traditions address, it is plausible that these cardiometabolic conditions may be correlated with some aspects of R/S and investigation of the relations between R/S, insulin resistance, and MetS could provide evidence regarding mechanisms associating some components of R/S and physical health.

We are aware of two studies that have investigated the possible relations of R/S and MetS. Both were conducted among middle-aged or older adults in the US. Brintz and colleagues [21] investigated the relations between religious activity and spiritual well-being with MetS among participants in the Hispanic Community Health Study/Study of Latinos Sociocultural Ancillary Study. Results demonstrated that neither religious activity nor spiritual well-being were significantly associated with the presence of MetS. Though some R/S aspects related to individual components of MetS, these associations were small in magnitude and largely disappeared when corrections for multiple comparisons were applied. The inverse association between faith and diastolic blood pressure was the only relationship that remained significant. A second study [34] focused on Hispanic women from the Study of Women’s Health Across the Nation (SWAN). Though, overall, Hispanic women had higher rates of incident MetS than non-Hispanic women, the investigators found that Hispanic women with high measures of religious faith had incidence rates lower than their low faith Hispanic counterparts and the incidence rates for those with high faith were similar to the non-Hispanic participants. The authors suggested that beneficial, health enhancing, aspects of R/S may play a role in the ‘Hispanic Paradox’, an unexpected finding in the literature that despite increased risk for adverse health outcomes due to poverty, lower education status, and inadequate health care coverage, Hispanic populations often have equal or better health outcomes and lower mortality when compared with non-Hispanics. Given the small number of investigations of R/S in relation to insulin resistance and MetS, and given the focus on Hispanic subpopulations, more research is needed on whether diverse aspects of R/S are related to cardiometabolic outcomes in the national population.

The aim of the current study was to further understanding of cross-sectional and prospective relations between multidimensional R/S and health outcomes by examining associations between religious service attendance and other, less studied, aspects of R/S with insulin resistance and MetS in a large, diverse sample of US adults. We hypothesized that greater endorsement of R/S, both in terms of service attendance and other dimensions, including religious identification, spirituality, R/S coping, private religious practices, daily spiritual experiences, R/S based mindfulness, and religious support, would be inversely related with measures of insulin resistance and MetS.

## Methods

### Sample

Data for this study came from the biomarker subsamples of the Midlife in the United States (MIDUS) study, a national longitudinal survey conducted among non-institutionalized, English-speaking midlife and older adults. In 1995–1996, wave 1 of MIDUS (M1) data were collected from N  =  7,108 respondents recruited via random-digit-dialing (RDD), siblings of RDD participants, and a national sample of twins. Between 2004 and 2006, a second wave of MIDUS data (M2) were collected, and 75% of M1 participants provided follow-up data, adjusting for mortality (N  =  4,968); [35]. At wave 2 of MIDUS (M2), a new sample of Black/African American adults from Milwaukee, Wisconsin was added to the study (N  =  592). During 2011-2014, a new sample of adults (i.e., Refresher cohort; MR) was recruited to match the gender and age distribution of participants from M1 (MR; N  =  3,577). At MR, an additional sample of Black and African American adults were recruited from Milwaukee (N  =  508). MIDUS data are publicly available at the Inter-University Consortium for Political and Social Research website, https://www.icpsr.umich.edu/web/DAIRL/series/203.

The analytic sample includes the subset of participants from the M2 (n  =  1,255; includes 16.0% Milwaukee respondents) and MR (n = 863; includes 13.6% Milwaukee respondents) cohorts that provided biological data. Participants in the biomarker subsamples travelled to one of three General Clinic Research Centers in the United States and stayed overnight for data collection. The response rates for participating in the biomarker sub-study were 43% for the M2 cohort (among main RDD sample and twins), 51% for the M2 Milwaukee respondents, 40% for the MR RDD cohort, and 67% for the MR Milwaukee cohort. Prior research in MIDUS showed that participant demographic and health characteristics were similar among the M2 biomarker subsample compared to the M2 survey sample who did not take part in the biomarker sub-study (i.e., age, gender, race, income, marital status, self-rated health, chronic conditions, body mass index (BMI), physician visits). However, the M2 biomarker subsample had higher levels of education and were less likely to smoke compared to the M2 survey sample [36]. Similarly, MR participants in the biomarker subsample were comparable to the MR survey sample who did not complete the biomarker sub-study for some characteristics (i.e., age, race, marital status, chronic conditions, BMI), however, they differed in that they were older, more educated, of higher socioeconomic status, less likely to smoke, had better self-rated health, and more frequent physician visits [37].

### Ethics statement

Data collection procedures were approved by the Institutional Review Boards at the University of Wisconsin-Madison, Georgetown University, and the University of California, Los Angeles. Written informed consent was obtained from all participants.

## Measures

### Religiousness and spirituality

At M1, three R/S scales were administered, including religious identification, spirituality, and religious/spiritual coping [38–40]. Religious identification was measured with six items, and a mean score was computed if participants provided data on at least three of the six items. Internal consistency for religious identification among the analytic sample was α = .90 at both M1 and M2/MR waves. Spirituality was measured using two items, and internal consistency was α = .91 at both M1 and M2/MR waves. Response options for both religious identification and spirituality ranged from 1 ‘very’ to 4 ‘not at all.’ Religious/spiritual coping (version A) was measured as the mean of two items and response options ranged from 1 ‘often’ to 4 ‘never.’ Internal consistency for religious/spiritual coping A was α = .88 at M1 and α = .87 at M2/MR. All items for the R/S scales at M1 are listed in S1 Appendix. All scales were recoded so that higher scores reflect higher R/S.

Additional R/S measures were added to the survey at M2 and MR, including private religious practices, a second measure of religious/spiritual coping, daily spiritual experiences, R/S based mindfulness, and religious support [41–45]. Private religious practices were measured using three items and response options ranged from 1 ‘once a day or more’ to 6 ‘never.’ Internal consistency for private religious practices was α = .71. Religious/spiritual coping (version B) included six items with response options ranging from 1 ‘A great deal’ to 4 ‘none;’ internal consistency was α = .65. Daily spiritual experiences included five items and response options ranged from 1 ‘often’ to 4 ‘never.’ Internal consistency was α = .94. R/S based mindfulness was measured using nine items. Response options ranged from 1 ‘strongly agree’ to 5 ‘strongly disagree,’ and internal consistency was α = .95. Religious support was measured with four items only among respondents who reported having a religious community or congregation (n =  761 for M2 and n =  445 for MR). Response options for religious support ranged from 1 ‘a great deal’ to 4 ‘none,’ and internal consistency was α = .59. All items for the R/S scales at M2/MR are listed in S1 Appendix. All scales were re-coded as necessary so that higher scores reflect higher R/S. Each scale was computed by calculating the sum across items in each scale and the mean of completed items was imputed when an item had a missing value [41,42].

Two principal components analyses of R/S scales were conducted at M1 and at M2/MR, respectively, to evaluate whether R/S measures could be consolidated [3,46,47]. At M1, one factor explained 77.5% of the variance in the religious identification, spirituality, and religious/spiritual coping (A) scales. All scales loaded highly on one factor (mean factor loading =  0.88; range: 0.86-0.91). At M2/MR, the principal components analysis included seven scales: religious identification, spirituality, religious/spiritual coping versions A & B, R/S based mindfulness, daily spiritual experiences, and private religious practice. All scales loaded highly on one factor (mean factor loading = .79; range: 0.63-.87), and one factor explained 63.7% of the variance. Because these R/S measures demonstrated considerable overlap, composite measures were computed for both M1 and M2/MR measures to minimize multiple comparisons. Both R/S composite variables were created by averaging standardized (i.e., z-scored to have a mean of 0 and standard deviation of 1) scales. The internal consistency ratings for both composite scales were α = .85. Because fewer participants completed the religious support measure, it was not included in the M2/MR R/S composite and was modeled as a separate z-scored predictor.

At M1, frequency of service attendance was measured with a single item (i.e., “How often do you usually attend religious or spiritual services?”). Response options included 1 ‘more than once a week,’ 2 ‘about once a week,’ 3 ‘one to three times a month,’ 4 ‘less than once a month,’ and 5 ‘never.’ In M2 and MR, this item was reworded to “Within your religious or spiritual tradition, how often do you attend religious or spiritual services?” and response options changed to 1 ‘once a day or more,’ 2 ‘a few times a week,’ 3 ‘once a week,’ 4 ‘1-3 times per month,’ 5 ‘less than once per month,’ 6 ‘never.’ For all analyses, response options were recoded into three categories: weekly or more (combined ‘more than once a week’ and ‘about once a week’ at M1 and combined ‘once a day or more,’ ‘a few times a week,’ and ‘once a week’ at M2/MR), less than weekly (combined ‘one to three times a month’ and ‘less than once per month’), and never (reference group).

### Insulin resistance

Measurement of insulin resistance was taken at MR and M2 using the homeostatic model assessment of insulin resistance (HOMA-IR) and was computed using the following formula: HOMA-IR =  (glucose *  insulin)/ 405 [48]. Fasting glucose (mg/dL) and insulin concentrations (μIU/mL) were derived from a fasting blood sample collected on the second day of the overnight clinic visit and assayed using standard procedures [49,50]. For descriptive purposes only, we adopted a cutoff of 2.7 to indicate problematic insulin resistance in Table 1. Importantly, there is no widely accepted threshold for problematic HOMA-IR levels [51,52], but 2.7 was used in prior research in the National Health and Nutrition Examination Survey [53]. In primary analyses, HOMA-IR was log-transformed to correct for a non-normal distribution. After z-scoring the log-transformed HOMA-IR variable, 16 extreme cases were observed (i.e., values outside of three standard deviations from the mean), and thus, were considered outliers and excluded from subsequent analyses. Sensitivity analyses demonstrated that exclusion of these 16 cases did not affect results.

**Table 1:** Descriptive information for MIDUS 1 and MIDUS 2/MIDUS Refresher cohorts in the analytic sample.

Variable^a^	M2/MR (n = 2,118)	M1 only^b^ (n = 1,054)
Age, in years	55.4 (12.6)	46.2 (11.8)
Sex (% female)	54.9% (1,163)	54.7% (577)
Race (% White)	74.5% (1,578)	93.8% (961)
Education		
% ≤ high school education	23.6% (499)	27.4% (289)
% Bachelor’s degree or higher	46.2% (977)	44.1% (464)
Marital status (% married/cohabitating)	62.6% (1,325)	71.3% (752)
Chronic conditions, count	2.6 (2.7)	2.2 (2.3)
Self-rated health (1 = poor, 5 = excellent)	3.6 (1.0)	3.7 (0.9)
Depressive symptoms (range = 0 to 7)	0.6 (1.8)	0.7 (1.9)
BMI	30.0 (7.1)	29.2 (6.0)
Diabetes status (% yes)	17.0% (360)	14.9% (154)
M1 Religious service attendance		
% weekly or more than weekly		42.3% (431)
% less than weekly		39.7% (404)
% never		18.0% (183)
M1 R/S composite		0.0 (0.8)
Religious/spiritual coping A (range = 1 to 4)		2.8 (1.1)
Spirituality (range = 1 to 4)		3.1 (0.8)
Religious identification (range = 1 to 4)		2.8 (0.8)
M2/MR Religious service attendance		
% weekly or more than weekly	43.4% (920)	
% less than weekly	31.3 (663)	
% never	24.4 (517)	
M2/MR R/S composite	0.0 (0.8)	
Mindfulness (range = 9 to 45)	34.2 (6.8)	
Daily spiritual experiences (range = 5 to 20)	15.8 (3.2)	
Religious/spiritual coping A (range = 2 to 8)	5.6 (2.2)	
Religious/spiritual coping B (range = 6 to 24)	18.3 (3.9)	
Spirituality (range = 2 to 8)	6.5 (1.7)	
Religious identification (range = 7 to 28)	19.4 (6.0)	
Private religious practice (range = 3 to 18)	9.8 (4.5)	
M2/MR Religious support (range = 5 to 16)	13.9 (1.8)	
Presence of MetS (1 = meets criteria)	33.3% (697)	34.6% (363)
Waist circumference – female (cm)	93.5 (17.9)	90.7 (14.8)
Waist circumference – male (cm)	103.7 (16.4)	104.1 (15.6)
Triglycerides (mg/dL)	123.3 (74.4)	130.6 (79.6)
Fasting glucose (mg/dL)	101.9 (27.4)	100.4 (24.8)
HDL cholesterol (mg/dL)	56.9 (18.7)	54.6 (17.6)
Systolic BP (mm Hg)	130.0 (17.9)	131.1 (17.7)
Diastolic BP (mm Hg)	76.5 (10.6)	75.0 (10.2)
Antihypertensive medications	8.4% (178)	8.3% (87)
Antidiabetic medications	10.5% (223)	8.7% (92)
Antihyperlipidemic medications	28.0% (593)	31.1% (327)
HOMA-IR	1.0 (.78)	0.9 (0.8)
Insulin (uIU/mL)	15.3 (17.1)	12.8 (12.3)
% HOMA-IR > 2.7	47.9%	40.5%

^a^Data are presented as *M*(*SD*) or % (n).

^b^All M1 variables were collected at wave 1, except for biological variables collected at the M2/MR biomarker visit (i.e., BMI, diabetes status, MetS criteria and incidence, HOMA-IR, insulin, and medications) Abbreviations: M1 =  MIDUS 1; M2 =  MIDUS 2; MR =  MIDUS Refresher; R/S =  religiousness/spirituality; BMI =  body mass index; HOMA-IR =  insulin resistance; MetS =  metabolic syndrome; HDL =  high-density lipoprotein; BP =  blood pressure. *Note*. HOMA-IR was log-transformed.

### Metabolic syndrome

MetS was assessed at M2 and MR based on the following five biological criteria: waist circumference, triglycerides, high density lipoprotein cholesterol (HDL), blood pressure, and glucose [54]. Triglycerides, HDL, and glucose were ascertained from a fasting blood sample taken on the second day of the overnight clinic visit. Blood pressure was measured by averaging the second and third of three total blood pressure measurements. Both waist circumference and blood pressure were measured by clinicians at the clinic visit. All biomarkers were assayed using standard protocols [36] and full descriptions of these procedures are publicly available [49,50]. Three triglyceride cases were coded as missing due to their values being outside of the specified assay range (i.e., values >  875 mg/dL). Based on cutoffs specified in Alberti et al. [54], a MetS symptom count was calculated based on the following criteria: waist circumference ≥ 94 cm for all non-Asian-identifying males, ≥ 90 cm for all Asian-identifying males, or ≥ 80 cm for all females; triglycerides ≥ 150 mg/dL; HDL < 40 mg/dL (males) or < 50 mg/dL (females); blood pressure ≥ 130 mmHg systolic or ≥ 85 mmHg diastolic or taking antihypertensive medication; fasting glucose ≥ 100 mg/dL or taking antidiabetic medication [55,56]. Participants were classified as having MetS if they met three or more criteria. If participants were missing data on three or more of the criteria, they were coded as missing.

### Covariates

Demographic factors included age (continuous in years), sex (0 ‘male,’ 1 ‘female), race (0 ‘Non-White’ [i.e., Black/African American, Native American or Aleutian Islander/Eskimo, Asian or Pacific Islander, Other], 1 ‘White’), educational attainment (continuous), and marital status (1 ‘married/cohabitating’, 0 ‘separated/divorced/widowed/never married). Models with M2/MR predictors also included sample (0 ‘MR’, 1 ‘M2’) as a covariate. Race was measured with the item, “Which best describes your race?” at M1 and the first response from the item, “What are your main racial origins - that is, what race or races are your parents, grandparents, and other ancestors?” at M2/MR. All participants from the M2 and MR Milwaukee subsamples were coded as ‘Non-White.’ Educational attainment responses ranged from 1 ‘no school or some grade school [1–6]’ to 12 ‘doctoral or other professional degree.’ All M1 demographic covariates were measured at wave 1. M2/MR sex, race, and educational attainment were included from wave 2 core and refresher surveys, and M2/MR age and marital status were included from the biomarker clinic visit [38,41,42].

Health status covariates included self-rated health (continuous), chronic conditions (continuous), and depressive symptoms (continuous) measured at both M1 and M2/MR survey waves. Self-rated health was measured with the item, “In general, would you say your physical health is excellent, very good, good, fair, or poor?” This variable was reverse coded so that higher scores reflect better self-rated health. Number of chronic conditions was measured by summing the total number of conditions that participants endorsed having experienced over the past 12 months (of 29 possible conditions). Depressive symptoms was computed based on seven items (e.g., “During two weeks in the past 12 months, when you felt sad, blue, or depressed, did you… ‘lose interest in most things?’”) [57]. Because self-reported physical health, chronic conditions, and depressive symptoms have been linked to R/S in prior literature [58,59], these measures were considered as potential confounds and were included as covariates in all models. When M1 R/S predictors were examined, M1 health status covariates were included and when M2/MR R/S predictors were examined, M2/MR health status covariates were included.

Additional covariates collected at the M2/MR biomarker visit were added to HOMA-IR and MetS models separately. Diabetes status (0 ‘no,’ 1 ‘yes’) and BMI were included in models predicting HOMA-IR. BMI was calculated by dividing participant weight (kilograms) by the square of their height (meters), both of which were measured by clinic staff. In models examining MetS, antihyperlipidemic medications were added as a covariate [55,56].

### Statistical analyses

To examine associations between R/S, HOMA-IR, and MetS, we conducted two sets of analyses. Key predictors were modeled separately and included M1 and M2/MR R/S composites, M1 and M2/MR religious service attendance, and M2/MR religious support, respectively. For M1 models, the analytic sample is smaller (n = 1,054) because there are no longitudinal data for the Milwaukee subsample. We include the M1 R/S data as a predictor of M2 health to assess whether prospective analyses align with cross-sectional associations reported for M2/MR. Missing data were limited to <  2% on any given variable, except for religious support. All analyses were run with complete case data, and all continuous variables were mean-centered.

First, ordinary least squares (OLS) regression models were run to examine associations between R/S measures and HOMA-IR. All models included demographic factors, self-rated health, chronic conditions, and depressive symptoms at either M1 or M2/MR (depending on when the key predictor was measured), as well as diabetes status and BMI at M2/MR. Second, logistic regression analyses were run to examine associations between R/S predictors and MetS. In addition to demographic factors, self-rated health, chronic conditions, and depressive symptoms, antihyperlipidemic medications were also controlled. In total, five models (two longitudinal and three cross-sectional models) were run predicting MetS and five models (two longitudinal and three cross-sectional models) were run predicting HOMA-IR. Exploratory analyses (alpha set at .01) examined whether the R/S measures interacted with gender in the prediction of HOMA-IR and MetS, and none of these interactions was significantly different from zero.

## Results

### Sample descriptives

Descriptive statistics are provided in Table 1. On average, participants were 55.4 years old, 54.9% female, 74.5% White, and 62.6% married or cohabitating. One-third of the analytic sample (33.3%) met criteria for MetS. Bivariate correlations are presented in Table 2. HOMA-IR was higher among male respondents, non-White respondents, those with lower educational attainment, lower self-rated health, greater number of chronic conditions, incident diabetes, and higher BMI. MetS was also more prevalent among males, those with lower educational attainment, lower self-rated health, and a greater number of chronic conditions.

**Table 2:** Bivariate correlations for all key variables.

Variable	2	3	4	5	6	7	8	9	10	11	12	13	14	15	16	17	18	19	20	21
1. M1 R/S Composite	.63**	-.19**	.82**	.59**	-.12**	.25**	.03	-.01	.07 *	.18**	-.04	-.04	.01	–	.00	.06*	-.03	.04	.01	-.11**
2. M1 Weekly attendance		-.70**	.53**	.67**	-.33**	.20**	.01	-.04	.13**	.04	-.01	.03	.12**	–	.03	-.03	-.08*	-.01	-.00	-.04
3. M1 < Weekly attendance			-.18**	-.38**	.41**	-.16**	-.01	.03	-.09*	.00	.01	-.01	-.07*	–	-.02	.04	.04	.03	.01	.02
4. M2/MR R/S Composite				.61**	-.12**	.35**	.00	.00	.09**	.23**	-.19**	-.09**	-.06*	.12**	-.03	.05*	-.01	.04*	.05*	-.06*
5. M2/MR Weekly attendance					-.60**	.25**	-.01	-.01	.10**	.09**	-.02	.03	.07**	.08**	.03	-.05*	-.08**	.01	.03	-.02
6. M2/MR < Weekly attendance						-.20**	.01	.01	-.04	-.03	-.02	-.02	-.03	-.00	-.02	.07*	.02	-.00	-.00	.01
7. M2/MR Religious support							-.00	-.01	.13**	.06	.07*	-.06*	.12**	.09*	.06*	-.04	-.10**	-.02	.01	-.03
8. HOMA-IR								.50**	-.02	-.11**	-.14**	-.15**	-.04	-.14**	-.28**	.18**	.05*	.52**	.39**	.21**
9. MetS diagnosis									.04	-.13**	-.03	-.10**	-.00	.04*	-.19**	.11**	.03	.34**	.29**	.12**
10. Age										-.06*	.20**	-.02	.05*	.18**	.01	.08**	-.13**	-.05*	.14**	.34**
11. Sex (female)											-.11**	-.08**	-.20**	.05*	-.04	.14**	.13**	.03	.01	-.14**
12. Race (white)												.22**	.33**	.09**	.25**	-.13**	-.05*	-.18**	-.17**	.10**
13. Education													.13**	-.13**	.24**	-.15**	-.09**	-.16**	-.15**	-.01
14. Marital status (married)														.05*	.17**	-.15**	-.10**	-.10**	-.08**	.09**
15. Sample (M2)															.02	-.06*	-.02	-.04*	.04	.04
16. Self-rated health																-.43**	-.21**	-.31**	-.25**	-.08**
17. Chronic conditions																	.26**	.19**	.22**	.08**
18. Depressive symptoms																		.11**	-.01	-.04*
19. BMI																			.27**	.11**
20. Incident diabetes																				.20**
21. Antihyperlipidemic medications																				

Abbreviations: M1 =  MIDUS 1; M2 =  MIDUS; R/S =  religiousness/spirituality; HOMA-IR =  insulin resistance; MetS =  metabolic syndrome; BMI =  body mass index. *Note.* All included demographic and health status covariates are from M2/MR survey waves.

* *p* < .05,

** *p* ≤ .001.

To investigate potential for selection bias based on R/S, we used independent samples t-tests and chi-square analyses to compare levels on all R/S variables between respondents in the biomarker sub-studies with respondents from M2 and MR survey samples who did not participate in the biomarker sub-studies. Results are presented in S2 Table. M2 biomarker study participants were lower on the R/S composite at wave 1 compared to respondents who did not participate in the biomarker study at wave 2. However, biomarker participants had higher wave 1 weekly service attendance relative to non-biomarker participants. There were no significant differences on the R/S composite or in service attendance between biomarker participants and non-participants at the M2/MR waves.

Bivariate correlations (Table 2) also demonstrated that the R/S composite at M1 was higher among participants who were older, female, among those who reported more chronic conditions, and among those not taking antihyperlipidemic medications. The R/S composite at M2/MR was similarly higher among older participants, females, non-White adults, those who were not married, those who had lower educational attainment, those with more chronic conditions, higher BMI, incident diabetes, and those not taking antihyperlipidemic medications. Neither R/S composite was significantly correlated with HOMA-IR and incident MetS. Weekly service attendance at M1 was correlated with older age, married/cohabitating status, and lower depressive symptoms. At M2/MR, weekly attendance was correlated with older age, female sex, married/cohabitating status, and fewer chronic conditions. Less than weekly service attendance was correlated with younger age and non-married status at M1, and with a greater number of chronic conditions and lower depressive symptoms at M2/MR. Service attendance at M1 and M2/MR was not correlated with either HOMA-IR or MetS. Finally, religious support at M2/MR was higher among participants who were older, White, married, and among those who had lower educational attainment, better self-rated health, and lower depressive symptoms. Religious support at M2/MR was not correlated with either HOMA-IR or MetS

R/S composites were highly correlated with each other (r = .82), and moderately correlated with weekly service attendance (r’s ≥ .53) and religious support (r’s ≥ .25). R/S composites were lower among those who attended religious services less than weekly compared to weekly attenders (r’s ≤  -.12).

### Religiousness/Spirituality and cardiometabolic outcomes

Table 3 provides results of linear regression analyses examining associations between R/S predictors and HOMA-IR. After controlling for age, sex, race, education, marital status, self-rated health, sample (M2 vs. MR), number of chronic conditions, depressive symptoms, BMI, and diabetes status, there were no significant associations observed between the R/S composites at M1 and M2/MR or M2/MR religious support and HOMA-IR. Further, there were no significant differences in levels of HOMA-IR between those who attended religious services less than weekly, or weekly or more, compared to those who never attended religious services. S3 Table shows results for all individual R/S scales predicting HOMA-IR. None of the individual scales were significantly associated with HOMA-IR in fully adjusted models.

**Table 3:** Linear regression results for R/S measures predicting HOMA-IR.

	Model 1 (Demographics)			Model 2 (Model 1 + Health Covariates)		
	B(SE)	*p*	95% CI	B(SE)	*p*	95% CI
M1 R/S Composite^a^	.05 (.03)	.09	-.01,.10	.03 (.02)	.24	-.02,.07
M1 ≥ Weekly service attendance (vs. never)^b^	.02 (.07)	.72	-.11,.15	-.01 (.05)	.90	-.11,.10
M1 < Weekly service attendance (vs. never)^b^	-.02 (.07)	.80	-.15,.11	-.07 (.05)	.19	-.17,.03
M2/MR R/S Composite^c^	.02 (.02)	.50	-.03,.06	.01 (.02)	.56	-.03,.05
M2/MR ≥ Weekly service attendance (vs. never)^d^	.03 (.04)	.56	-.06,.11	.01 (.04)	.80	-.06,.08
M2/MR < Weekly service attendance (vs. never)^d^	.01 (.05)	.87	-.08,.10	.00 (.04)	.99	-.07,.07
M2/MR Religious support^e^	.02 (.02)	.73	-.03,.06	.02 (.02)	.25	-.02,.06

Abbreviations: R/S – religiousness/spirituality; HOMA-IR – insulin resistance; M1 =  MIDUS 1; M2 =  MIDUS 2; MR =  MIDUS Refresher. *Note.* HOMA-IR was log-transformed. Model 1 included age, sex, race, education, marital status, and sample (M2 vs. MR). Model 2 included Model 1 covariates plus self-rated health, chronic conditions, depressive symptoms, body mass index, and diabetes status.

^a^Sample size is 992;

^b^sample size is 988;

^c^sample size is 2,026;

^d^sample size is 2,019;

^e^sample size is 1,156.

Table 4 provides results of logistic regression analyses examining associations between R/S predictors and incident MetS. After controlling for demographic factors, self-rated health, sample, chronic conditions, depressive symptoms, and antihyperlipidemic medications, no significant associations were observed between the R/S composites at M1 and M2/MR R/S or M2/MR religious support and MetS incidence. Additionally, the likelihood of having MetS was not associated with attending religious services less than weekly, or weekly or more, compared to those who never attend religious services across both M1 and M2/MR. S4 Table shows results for all individual R/S scales predicting MetS. None of the individual scales were significantly associated with MetS.

**Table 4:** Logistic regression results for R/S measures predicting MetS diagnosis.

	Model 1 (Demographics)			Model 2 (Model 1 + Health Covariates)			Model 3 (Model 2 + Medication)		
	B(SE)	*p*	OR	B(SE)	*p*	OR	B(SE)	*p*	OR
M1 R/S Composite^a^	.07 (.08)	.42	1.07	.07 (.08)	.38	1.07	.11 (.08)	.19	1.11
M1 ≥ Weekly service attendance (vs. never)^b^	-.03 (.20)	.89	.97	.02 (.20)	.93	1.02	.06 (.20)	.77	1.06
M1 < Weekly service attendance (vs. never)^b^	.11 (.20)	.57	1.12	.14 (.20)	.47	1.15	.15 (.20)	.45	1.16
M2/MR R/S Composite^c^	.06 (.06)	.35	1.06	.08 (.07)	.25	1.08	.09 (.07)	.15	1.10
M2 ≥ Weekly service attendance (vs. never)^d^	-.00 (.12)	.99	1.00	.03 (.13)	.80	1.03	.06 (.13)	.66	1.06
M2 < Weekly service attendance (vs. never)^d^	-.00 (.13)	.98	1.00	-.01(.13)	.93	1.00	-.01(.13)	.97	0.99
M2 Religious support^e^	-.01 (.07)	.94	1.00	.03 (.07)	.66	1.03	.04 (.07)	.53	1.04

Abbreviations: R/S – religiousness/spirituality; MetS – metabolic syndrome; M1 =  MIDUS 1; M2 =  MIDUS 2; MR =  MIDUS Refresher. *Note.* All continuous covariates were mean-centered. Model 1 included age, sex, race, education, marital status, and sample (M2 vs. MR). Model 2 included Model 1 covariates plus self-rated health, chronic conditions, and depressive symptoms. Model 3 included Model 2 covariates plus antihyperlipidemic medications.

^a^Sample size is 1,008;

^b^sample size is 1,004;

^c^sample size is 2,063;

^d^sample size is 2,056;

^e^sample size is 1,179.

## Discussion

This study was designed to further understanding of the processes that may account for relations previously observed between R/S and morbidity and mortality risk. Specifically, we used the MIDUS dataset to interrogate several components of R/S, operationalized through religious service attendance and several other less explored, yet possibly potent, R/S variables including religious identification, spirituality, R/S coping, private religious practices, daily spiritual experiences, R/S based mindfulness, and religious support. To investigate physiological processes that may influence morbidity and mortality, and may be influenced by R/S variables, we studied insulin resistance and MetS. Both are subclinical pathways to poor health outcomes including T2DM, cardiovascular diseases, and mortality [31–33].

Our findings indicate that in both cross-sectional and longitudinal analyses, using bivariate correlations and adjusted regression models, no significant relations were found between any measures of R/S (measured as composite variables or as individual scales) and insulin resistance or MetS. Thus, though both insulin resistance and MetS are known to be influenced by lifestyle and psychological factors, and both seemed to be promising targets of investigation, our study failed to support hypotheses that greater endorsement of these aspects of R/S would be inversely related with measures of insulin resistance and MetS. These findings need to be contextualized within the limited literature specific to these variables as well as within the larger literature examining possible mechanisms in the R/S and health relationships.

Brintz et al. [21] conducted a cross-sectional study examining the relations between dimensions of both organizational and non-organizational R/S as well as spiritual well-being with MetS in a diverse Hispanic/Latino sample. They also found largely null relations. Alternatively, in a seven-year longitudinal study of Hispanic women, Allshouse et al [34] found that multidimensional assessment of R/S predicted lower incidence of MetS for Hispanic women characterized by higher measures of R/S than for those with lower measures of R/S. Further, incidence rates for those higher in R/S were similar to rates found for non-Hispanic participants in the parent study, eliminating the health disparity for MetS. As noted, our study included both cross-sectional and longitudinal assessments with both failing to find significant relations between R/S and MetS, replicating the Brintz et al. findings but failing to sustain those of Allshouse et al. The present study differed from both previous ones in that they were conducted with Hispanic samples (in one case all female) whereas the present study included a more diverse sample that was only about 4% Hispanic.

Another area of exploration concerns the operationalization and measurement of R/S. In particular, the primary predictor in Allshouse et al. [34] that demonstrated beneficial longitudinal relations with MetS was an item assessing agreement with “my faith sustains me,” wherein those with greater agreement had lower incidence of MetS. This provides a measure of the function of faith, i.e., what it is that faith does in an individual’s life. Alternatively, the R/S operationalization in our study combined both functional and structural R/S measures, via data reduction through factor analysis, into R/S composites alongside service attendance and religious support. Differences between these operationalizations of R/S may account for at least some of the disparate findings, although supplemental analyses show that associations did not differ appreciably when R/S scales were considered individually. Allshouse et al. noted that the Hispanic women in their study demonstrated more strength and comfort from spirituality, more frequent prayer, and being more sustained by faith than did the non-Hispanic women. Similarly, Shattuck and Muehlenbein [22] pointed out that prayer and meditation (considered by them aspects of intrinsic religiosity) demonstrated some of the stronger relations with health outcomes in their meta-analysis and they further suggest that some aspects of R/S may have physiological effects via anxiolytic (e.g., strength and comfort) properties.

Previous investigations show that greater levels of several R/S components are related to “healthier” lifestyle profiles that include lower rates of smoking or illegal drug use, moderate or no use of alcohol, increased physical activity, increased use of preventive health services, greater meaning/purpose in life, improved stress management, and greater overall self-regulatory ability [27], although not all studies support these patterns [23,24]. This lifestyle pattern is, hypothetically, suggestive of improved cardiometabolic function and therefore reduced insulin resistance and MetS, thus nominating these variables as prominent potential physiological pathways through which R/S may influence health outcomes. Further, Shattuck and Muehlenbein’s [22] meta-analysis on some R/S components and physiological markers of health indicated that some aspects of R/S were associated with several health-related biological markers including reduced blood pressure and cholesterol (two components of MetS). Our data, however, do not support these conclusions. One consideration is prior evidence from a review of quantitative studies on religion and body weight that found that nearly 70% of studies reported significant relations between greater religiosity and higher body weight [24]. Given the importance of obesity for insulin resistance and MetS [60], this may have affected our results. Table 2 shows that BMI was positively correlated with the R/S composite in cross-sectional models, but it was very small in magnitude and BMI was not associated with any other R/S measures in the analytic sample. Interestingly, Shattuck and Muehlenbein also found that a variable called “overall religiosity” that was not clearly defined but may be similar to our R/S composite variables, was the exception to their general findings regarding R/S variables being related to positive health outcomes (e.g., lower body mass index, lower inflammatory markers). These authors also noted that though they found a consistent pattern of results indicating some aspects of R/S were associated with better biological function, the effect sizes were generally small and inter-study heterogeneity was generally high. Identification of the mediators between R/S components and health outcomes remains an area ripe for investigation that currently lacks consensus [10,61]. Indeed, earlier work in the MIDUS 2 biological sample demonstrated that psychological well-being, including life satisfaction, positive relations with others, and purpose in life, predicted lower risk for metabolic syndrome [62], and many midlife and older adults identify R/S as the source of their psychological well-being [63–67].

Finally, we advanced research in this area by measuring R/S multidimensionally, resulting in two overall composites of related R/S variables plus service attendance and religious support. Investigators into the relations between R/S and health outcomes note that both R/S and health are multidimensional constructs worthy of incorporation of a plurality of relevant measures. Nevertheless, our methods indicated substantial relations among various R/S measures that resulted in two composite variables (from two different MIDUS timepoints) that both accounted for large amounts of variance, resulted in strong mean factor loadings, and had robust internal consistency. Consequently, we did not find support that the multidimensionality of R/S mattered for predicting physical health outcomes in this study (i.e., R/S composite variables and individual R/S scales did not show associations with MetS and HOMA-IR). Future research should continue to operationalize multiple dimensions of R/S to refine our understanding of the myriad ways that R/S matters in people’s lives and may also affect physical health. Prior research has shown that some people turn to religiosity and/or spirituality when coping with health challenges [68], and these patterns may obscure salubrious relationships among some aspects of R/S and health outcomes in the general population.

Our study has several strengths and limitations. First, drawing firm conclusions about the absence of an association between R/S and cardiometabolic biomarkers is challenging. We cannot rule out sampling or measurement errors as explanations for the lack of an observed association between R/S and HOMA-IR and MetS among midlife adults. However, the observed null associations were found in two distinct samples, with two unique outcomes, in both cross-sectional and prospective models, and with R/S measured multidimensionally, lending credence to their validity. Despite the large number of analyses run, we did not adjust for multiple comparisons, although conclusions regarding the lack of associations would be unchanged with this adjustment. There are limited investigations on multidimensional R/S and physiological biomarkers in the published literature, perhaps due to “file drawer” effects which align with the present results. Another consideration is that the biomarker samples, especially the Refresher sample, were significantly healthier and better educated than the MIDUS survey samples [36,37], and there was limited racial/ethnic heterogeneity within MIDUS. It is possible that a more representative sample would have more variability in these health outcomes. MIDUS 2 biomarker study participants were lower on the religiosity composite and more likely to be weekly service attenders compared to survey participants at the first wave who did not participate in the wave 2 biomarker sub-study (although there were no differences between biomarker participants and survey participants on R/S measured at wave 2 and the Refresher waves). Though it is unclear how these selection effects may have impacted the obtained results, it is important to acknowledge that the analytic sample does not represent the general U.S. adult population. Third, while several aspects of R/S were included in this study, key dimensions of R/S for health were not included (e.g., religious orientation; denomination; life meaning tied to R/S). Of these, religious orientation, which captures how individuals relate to their religious and/or spiritual beliefs and underlying motivations for R/S behaviors, may be particularly important to study given that prior research has shown that both mental and physical health are differentially predicted by intrinsic and extrinsic religious orientations [69,70]. Finally, given recent declines in religious affiliation in the United States, it is important to contextualize these results to the periods and cohorts during which data were collected. Future research should examine these important population health questions with more recent cohorts and more representative samples. Stronger associations may have been observed if a more representative sample with greater heterogeneity was investigated.

Mechanisms accounting for the associations between R/S and morbidity and mortality risk remain to be thoroughly identified. In a large sample of US adults, studied both cross-sectionally and prospectively, our study failed to support insulin resistance and MetS as possible physiological mechanisms in the path between R/S and health outcomes. Additional physiological mechanisms, such as chronic inflammation and cardiovascular reactivity to stress [71–73], may be more sensitive to variation in R/S. Consequently, this is an area that remains ripe for further investigation.

## Supporting information

S1 Appendix MIDUS religiosity and spirituality (R/S) scale items. (DOCX)

S2 Table Comparing M2/MR biomarker respondents with survey respondents who did not complete biomarker sub-study. (DOCX)

S3 Table Linear regression results for R/S measures predicting HOMA-IR. (DOCX)

S4 Table Logistic regression results for R/S measures predicting MetS diagnosis. (DOCX)
