# Supplementary material for: Associations between religiosity/spirituality with insulin resistance and metabolic syndrome in the Midlife in the United States (MIDUS) study
Source: PLoS One. 2025 Feb 21;20(2):e0319002. doi: 10.1371/journal.pone.0319002 (PMC11844912; doi:10.1371/journal.pone.0319002)
Supplement: S1 Appendix — (DOCX) [file pone.0319002.s001.docx]

**S1 Appendix. MIDUS religiosity and spirituality (R/S) scale items.**

**MIDUS 1 R/S Scales**

A. Religious Identification

1. “How religious are you?”

2. “How important is religion in your life?”

3. “How important is it for you -- or would it be if you had children now -- to send your children for religious or spiritual services or instruction?”

4. “How closely do you identify with being a member of your religious group?”

5. “How much do you prefer to be with other people who are the same religion as you?”

6. “How important do you think it is for people of your religion to marry other people who are the same religion?”

B. Spirituality

1. “How spiritual are you?”

2. “How important is spirituality in your life?”

C. Religious/Spiritual Coping (version A)

1. “When you have problems or difficulties in your family, work, or personal life, how attending a religious or spiritual service, or talking to a religious or spiritual advisor?”

2. “When you have decisions to make in your daily life, how often do you ask yourself what your religious or spiritual beliefs suggest you should do?”

**MIDUS 2 and MIDUS Refresher R/S Scales**

A. Private Religious Practices

(How often do you)

1. “Pray in private?”

2. “Meditate or chant?”

3. “Read the Bible or other religious literature?”

B. Religious/Spiritual Coping (version B)

1. “I try to make sense of the situation and decide what to do without relying on God” (R)

2. “I wonder whether God has abandoned me.” (R)

3. “I feel God is punishing me for my sins or lack of spirituality.” (R)

4. “I look to God for strength, support, and guidance.”

5. “I work together with God as partners.”

6. “I think about how my life is part of a larger spiritual force.”

C. Daily Spiritual Experiences

(On a daily basis, how often do you experience the following)

1. “A feeling of deep inner peace or harmony.”

2. “A feeling of being deeply moved by the beauty of life.”

3. “A feeling of strong connection to all of life.”

4. “A sense of deep appreciation.”

5. “A profound sense of caring for others.”

D. Religious/Spirituality-based Mindfulness

(Because of your religion or spirituality, do you try to be..)

1. “more engaged in the present moment.”

2. “more sensitive to the feelings of others.”

3. “more receptive to new ideas.”

4. “a better listener.”

5. “a more patient person.”

6. “more aware of small changes in my environment.”

7. “more tolerant of differences.”

8. “more aware of different ways to solve problems.”

9. “more likely to perceive things in new ways.”

E. Religious Support

1. “If you were ill, how much would people in your congregation help you out?”

2. “If you had a problem or were faced with a difficult situation, how much comfort would people in your congregation be willing to give you?”

3. “How often do people in your congregation or spiritual community make too many demands on you?” (R)

4. “How often do people in your congregation or spiritual community criticize you and the things you do?” (R)

*Note*. R = item is reverse coded for scale calculation. The scales detailed at MIDUS 1 were also administered at MIDUS 2 and MIDUS Refresher time points. Details (e.g., scale calculation rules, internal consistency, citations) regarding all psychosocial constructs and scales found in the Midlife in the United States (MIDUS) Study are documented in [1–3]

References

1. Brim OG, Baltes PB, Bumpass LL, Cleary PD, Featherman DL, Hazzard WR, et al. Midlife in the United States (MIDUS 1), 1995-1996: Version 19. ICPSR - Interuniversity Consortium for Political and Social Research; 1999. doi:10.3886/ICPSR02760.V19

2. Ryff CD, Almeida DM, Ayanian JZ, Carr DS, Cleary PD, Coe C, et al. Documentation of Psychosocial Constructs and Composite Variables in MIDUS 2. Available: https://www.icpsr.umich.edu/web/ICPSR/studies/4652/datadocumentation#

3. Ryff C, Almeida D, Ayanian J, Binkley N, Carr D, Coe C, et al. MIDUS Refresher Psychosocial Constructs and Composite Variables. Report No.: 36532.
