# Supplementary material for: Associations between religiosity/spirituality with insulin resistance and metabolic syndrome in the Midlife in the United States (MIDUS) study
Source: PLoS One. 2025 Feb 21;20(2):e0319002. doi: 10.1371/journal.pone.0319002 (PMC11844912; doi:10.1371/journal.pone.0319002)
Supplement: S2 Table — (DOCX) [file pone.0319002.s002.docx]

**S2 Table. Comparing M2/MR biomarker respondents with survey respondents who did not complete biomarker sub-study.**

|  | M2/MR biomarker | No M2/MR biomarker participation | *t* or Χ^2^ for difference |
| --- | --- | --- | --- |
| M1 RS Composite, *M*(*SD*)^a^ | -.17(.72) | .03(.98) | 6.35*** |
| M1 service attendance^b^ |  |  | 6.98* |
| ≥ Weekly attendance, % | 42.3% | 38.1% |  |
| < Weekly attendance, % | 39.7% | 41.4% |  |
| Never attend, % | 18.0% | 20.5% |  |
| M2/MR RSC, *M*(*SD*)^c^ | .01(.80) | -.002(.79) | 0.81 |
| M2/MR attendance^d^ |  |  | 1.92 |
| ≥ Weekly attendance, % | 43.8% | 42.2% |  |
| < Weekly attendance, % | 31.6% | 33.1% |  |
| Never attend, % | 24.6% | 24.6% |  |
| M2/MR religious support, *M*(*SD*)^e^ | 13.9(1.8) | 13.8(1.9) | 1.68 |

****p* < .001, ***p* < .01, **p* < .05

^a^*N*=1,051 for biomarker participants; *N*=5,989 for non-biomarker participants.

^b^*N*=1,017 for biomarker participants; *N*=5,207 for non-biomarker participants.

^c^*N*=2,107 for biomarker participants; *N*=5,190 for non-biomarker participants.

^d^*N*=2,099 for biomarker participants; *N*=5,139 for non-biomarker participants.

^e^*N*=1,205 for biomarker participants; *N*=3,010 for non-biomarker participants.
