# Supplementary material for: Associations between religiosity/spirituality with insulin resistance and metabolic syndrome in the Midlife in the United States (MIDUS) study
Source: PLoS One. 2025 Feb 21;20(2):e0319002. doi: 10.1371/journal.pone.0319002 (PMC11844912; doi:10.1371/journal.pone.0319002)
Supplement: S3 Table — (DOCX) [file pone.0319002.s003.docx]

**S3 Table. Linear regression results for R/S measures predicting HOMA-IR.**

|  | Model 1 (Demographics) | | | Model 2 (Model 1 + Health Covariates) | | |
| --- | --- | --- | --- | --- | --- | --- |
|  | **B(SE)** | ***p*** | **95% CI** | **B(SE)** | ***p*** | **95% CI** |
| M1 Religious Identification | .04(.03) | .17 | -.02, .10 | .01(.02) | .54 | -.03, .06 |
| M1 Spirituality | .05(.03) | .13 | -.01, .10 | .04(.02) | .095 | -.01, .08 |
| M1 R/S Coping (version A) | .04(.02) | .12 | -.01, .08 | .02(.02) | .39 | -.02, .05 |
| M2/MR Religious Identification | .01(.003) | .021 | .001, .01 | .004(.002) | .12 | -.001, .01 |
| M2/MR Spirituality | .001(.01) | .91 | -.02, .02 | .001(.01) | .87 | -.02, .02 |
| M2/MR R/S Coping (version A) | .01(.01) | .20 | -.01, .03 | .01(.01) | .49 | -.01, .02 |
| M2/MR Private Religious Practices | .003(.004) | .51 | -.01, .01 | -.001(.003) | .72 | -.01, .01 |
| M2/MR Daily Spiritual Experiences | -.003(.01) | .64 | -.01, .01 | .003(.004) | .54 | -.01, .01 |
| M2/MR Mindfulness | .00(.003) | .87 | -.01, .01 | .001(.002) | .54 | -.003, .01 |
| M2/MR R/S Coping (version B) | -.001(.004) | .75 | -.01, .01 | .00(.004) | .96 | -.01, .01 |

Abbreviations: R/S – religiousness/spirituality; HOMA-IR – insulin resistance; M1 = MIDUS 1; M2 = MIDUS 2; MR = MIDUS Refresher. *Note.* HOMA-IR was log-transformed. Model 1 included age, sex, race, education, marital status, and sample (M2 vs. MR). Model 2 included Model 1 covariates plus self-rated health, chronic conditions, depressive symptoms, body mass index, and diabetes status.
