# Supplementary material for: Associations between religiosity/spirituality with insulin resistance and metabolic syndrome in the Midlife in the United States (MIDUS) study
Source: PLoS One. 2025 Feb 21;20(2):e0319002. doi: 10.1371/journal.pone.0319002 (PMC11844912; doi:10.1371/journal.pone.0319002)
Supplement: S4 Table — (DOCX) [file pone.0319002.s004.docx]

**S4 Table. Logistic regression results for R/S measures predicting MetS diagnosis.**

|  | Model 1 (Demographics) | | | Model 2 (Model 1 + Health Covariates) | | | Model 3 (Model 2 + Medication) | | |
| --- | --- | --- | --- | --- | --- | --- | --- | --- | --- |
|  | **B(SE)** | ***p*** | **OR** | **B(SE)** | ***p*** | **OR** | **B(SE)** | ***p*** | **OR** |
| M1 Religious Identification | .08(.09) | .33 | 1.09 | .10(.09) | .24 | 1.11 | .14(.09) | .13 | 1.15 |
| M1 Spirituality | .02(.09) | .85 | 1.02 | .02(.09) | .80 | 1.02 | .06(.09) | .51 | 1.06 |
| M1 R/S Coping (version A) | .06(.07) | .35 | 1.06 | .06(.07) | .39 | 1.06 | .08(.07) | .24 | 1.08 |
| M2/MR Religious Identification | .01(.01) | .55 | 1.01 | .01(.01) | .59 | 1.01 | .01(.01) | .48 | 1.01 |
| M2/MR Spirituality | .001(.03) | .96 | 1.00 | .004(.03) | .91 | 1.00 | .01(.03) | .73 | 1.01 |
| M2/MR R/S Coping (version A) | .04(.02) | .071 | 1.04 | .04(.02) | .081 | 1.04 | .05(.02) | .045 | 1.05 |
| M2/MR Private Religious Practices | .01(.01) | .26 | 1.01 | .01(.01) | .42 | 1.01 | .01(.01) | .29 | 1.01 |
| M2/MR Daily Spiritual Experiences | .01(.02) | .50 | 1.01 | .02(.02) | .18 | 1.02 | .03(.02) | .11 | 1.03 |
| M2/MR Mindfulness | .001(.01) | .87 | 1.00 | .004(.01) | .55 | 1.00 | .01(.01) | .45 | 1.01 |
| M2/MR R/S Coping (version B) | .01(.01) | .54 | 1.01 | .02(.01) | .25 | 1.02 | .02(.01) | .17 | 1.02 |

Abbreviations: R/S – religiousness/spirituality; MetS – metabolic syndrome; M1 = MIDUS 1; M2 = MIDUS 2; MR = MIDUS Refresher. *Note.* All continuous covariates were mean-centered. Model 1 included age, sex, race, education, marital status, and sample (M2 vs. MR). Model 2 included Model 1 covariates plus self-rated health, chronic conditions, and depressive symptoms. Model 3 included Model 2 covariates plus antihyperlipidemic medications.
